# Anisocoria after use of dermatological product

**DOI:** 10.1590/S1679-45082017AI4040

**Published:** 2017

**Authors:** Thiago Gonçalves dos Santos Martins, Ana Luiza Fontes de Azevedo Costa, Thomaz Gonçalves dos Santos Martins

**Affiliations:** 1 Universidade Federal de São Paulo, São Paulo, SP, Brazil.; 2 Hospital das Clínicas, Faculdade de Medicina, Universidade de São Paulo, São Paulo, SP, Brazil.; 3 Universidade Estácio de Sá, Rio de Janeiro, RJ, Brazil.

**Figure 1:**
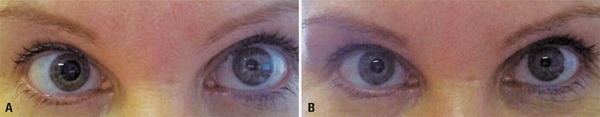
Patient with anisocoria after use cosmetic product and isocoric after discontinuing the use of the product

A 45-year-old woman sought ophthalmologic care complaining about a sudden visual acuity on her right eye and anisocoria. She denied previous comorbidities and traumas. Upon examination, her ocular motility was normal and no ptosis was seen. Slit-lamp biomicroscopy and fundoscopy exams were normal. The mydriasis of the right eye, with consensual light reflex of the left eye, did not show changes ( Figure 1 ). The patient, when questioned about the use of medication, reported the use on her face, in the night before the consultation, of a cosmetic jelly containing coffee extract in its composition. She was advice to discontinue the use of the jelly and, after 24 hours there was an improvement of the clinical picture.

Caffeine is a methylxanthine-derived alkaloid found in a variety of drinks that cause sympathetic release of norepinephrine that generate mydriasis, but this problem occurs only in patients who do not drink coffee regularly.^(^ ^1^ ^,^ ^2^ ^)^ The association of caffeine with increase of blood pressure and reduction brain blood flow has been shown.^(^ ^1^ ^,^ ^2^ ^)^ Caffeine has been also related with changes in retina and choroid, intraocular and pupil pressure.^(^ ^3^ ^,^ ^4^ ^)^

Sudden onset of anisocoria can indicate intracranial disease, but the chance of inappropriate use of mydriatic drugs must always be considered.^(^ ^5^ ^)^ A mydriatic pupil, unresponsive to light, can suggest Adie’s tonic pupil, affecting the third cranial nerve or pharmacological block. Posterior synechiae can also lead to this clinical picture after uveitis episode.

The pilocarpine 1% to test pupil reaction is a parasympathomimetic drug that can be used as a differential diagnosis for paralytic mydriasis and pharmacological block of iris sphincter muscle. The pupil contraction is observed in cases of paralytic mydriasis because of post-ganglionar nervous injury. Pharmacological mydriasis and traumatic mydriais do not respond to pilocarpine eye drop.^(^ ^6^ ^,^ ^7^ ^)^

Our report suggests that components of coffee extract can affect temporary the diameter of pupil when used topic in some patients. Patients should be advised about this possible effect.
